# Combining interleukin 6 and EBV DNA levels predicts survival outcomes for patients with recurrent or metastatic nasopharyngeal carcinoma receiving chemoimmunotherapy

**DOI:** 10.3389/fimmu.2025.1560897

**Published:** 2025-03-14

**Authors:** Ya Liu, Zilu Huang, Chen Chen, Yujun Hu, Yalan Tao, Songran Liu, Ping Feng, Shuohan Zheng, Yunfei Xia

**Affiliations:** ^1^ State Key Laboratory of Oncology in South China, Guangdong Key Laboratory of Nasopharyngeal Carcinoma Diagnosis and Therapy, Guangdong Provincial Clinical Research Center for Cancer, Sun Yat-sen University Cancer Center, Guangzhou, China; ^2^ Department of Radiation Oncology, Sun Yat-sen University Cancer Center, Guangzhou, China; ^3^ Department of Radiology, Sun Yat-sen University Cancer Center, Guangzhou, China; ^4^ Department of Pathology, Sun Yat-sen University Cancer Center, Guangzhou, China; ^5^ Department of Oncology, The Second Affiliated Hospital of Hengyang Medical School, Hengyang, Hunan, China

**Keywords:** interleukin-6, EBV DNA, prognostic risk grouping model, recurrent or metastatic nasopharyngeal carcinoma, chemoimmunotherapy

## Abstract

**Purpose:**

Platinum-based chemotherapy plus PD-1 inhibitors (chemoimmunotherapy) was the standard systemic treatment for recurrent or metastatic nasopharyngeal carcinoma (R/M NPC). However, biomarkers to predict the survival outcomes remained unsatisfying. This study aimed to establish a simple but easily applicable model to predict the survival outcomes of R/M NPC receiving chemoimmunotherapy.

**Materials and methods:**

A total of 319 R/M NPC patients treated by chemoimmunotherapy with or without local therapy at our hospital were randomly divided into training (n=223) and validation (n=96) cohorts at a ratio of 7:3. An easily applicable prognostic risk grouping model was created using common independent predictors of progression-free survival (PFS) and overall survival (OS) in the training set. Model performance was assessed in the validation set.

**Results:**

Pretreatment IL-6 and EBV DNA levels were identified as independent prognostic factors (scored on 0-4 points), and used to develop a prognostic risk grouping model with distinct survivals: 0-1 point (low risk), 2-3 points (intermediate risk), and 4 points (high risk). In the training set, the median PFS were not reached (NR), 18.90, and 7.73 months (P<0.001) respectively in the low-, intermediate-, and high-risk groups, while the median OS were NR, NR and 13.6 months (P<0.001). Results were further confirmed in the validation set.

**Conclusion:**

This model predicted both PFS and OS in R/M NPC patients undergoing chemoimmunotherapy. This finding may help clinicians with an initial prognostic estimation but warrants further prospective investigation for the value of IL-6 and EBV DNA.

## Introduction

Nasopharyngeal carcinoma (NPC), a malignancy originating from the nasopharynx epithelium, is endemic in southern China and Southeast Asia (1). Platinum-based concurrent chemoradiotherapy (CRT) has significantly improved survival outcomes for primary non-metastatic NPC (2). However, 5%-15% of NPC patients still experienced locoregional failure, and 15%-30% developed distant metastases after definitive chemoradiotherapy (3, 4). Furthermore, 4-10% presented with metastatic disease at the time of diagnosis (1, 3, 5). Currently, programmed death receptor antagonist 1 (PD-1) monoclonal antibody (PD-1 mAb), combined with cisplatin-based doublet or triplet chemotherapy regimens, have become the standard first-line treatment for recurrent or metastatic nasopharyngeal carcinoma (R/M NPC) (6–12). Despite the efficacy, the median progression-free survival (PFS) of patients treated with chemoimmunotherapy ranged from 9.6-21.4 months (7, 9, 12). This variability highlights the urgent need for reliable biomarkers or predictive indicators to identify patients who are most likely to benefit from chemoimmunotherapy.

Although nonkeratinizing NPC is characterized by a rich lymphocytic infiltration and high programmed death ligand 1 (PD-L1) expression in tumor tissue, the expression levels of PD-1/PD-L1 was limited to predict the efficacy of immunotherapy (13–17). Other ICIs-associated markers, such as microsatellite instability (MSI) and tumor mutational burden (TMB), were also difficult to identify patients who could benefit from immunotherapy (18). Genetic mutation analyses have identified several altered genes, including TP53, CDKN2A, and CDKN2B, but none have demonstrated sufficient predictive value for clinical efficacy (18, 19). Similarly, the density of MHC-II+ cell remains inadequate as a predictive factor (20). Inflammatory factors, like C-response protein (CRP), white blood cells (WBC), and neutrophils (NE) in the tumor or immune microenvironment, were demonstrated to play an important role in promoting tumor progression (19, 21, 22), but they were susceptible to interfering with treatment (like chemotherapy). To date, simple and reliable biomarkers for predicting the survival outcomes of R/M NPC patients undergoing chemoimmunotherapy remain unsatisfactory (18, 23).

Epstein-Barr virus (EBV) DNA, a stable biomarker, has been shown to be useful in predicting the treatment efficacy and monitoring disease progression in NPC patients (18, 24). However, its prognostic value in R/M NPC patients receiving chemoimmunotherapy was uncertain. Interleukin-6 (IL-6), an inflammation cytokine, was associated with cytotoxic T-cell (CTL) differentiation. Blocking the IL-6 receptor has been demonstrated to enhance the efficacy of immunotherapy by promoting tumor immunity while reducing toxicity (25–28). Recent results from prospective clinical trials have also indicated that elevated IL-6 levels were related to tumor progression and poor survival outcomes in patients undergoing ICIs for colon cancer or advanced non–small cell lung cancer (28–30). However, the relationship between IL-6 levels and the prognosis of NPC is unclear, particularly in R/M NPC patients receiving chemoimmunotherapy.

Therefore, our study aimed to evaluate whether pretreatment IL-6 levels could be a prognostic factor for R/M NPC. If possible, we want to combine EBV DNA and IL-6 levels to develop a simple but useful prognostic risk grouping model for R/M NPC receiving PD-1 mAb and platinum-containing chemotherapy.

## Materials and methods

### Data collection and patient enrollment

**Figure 1:**
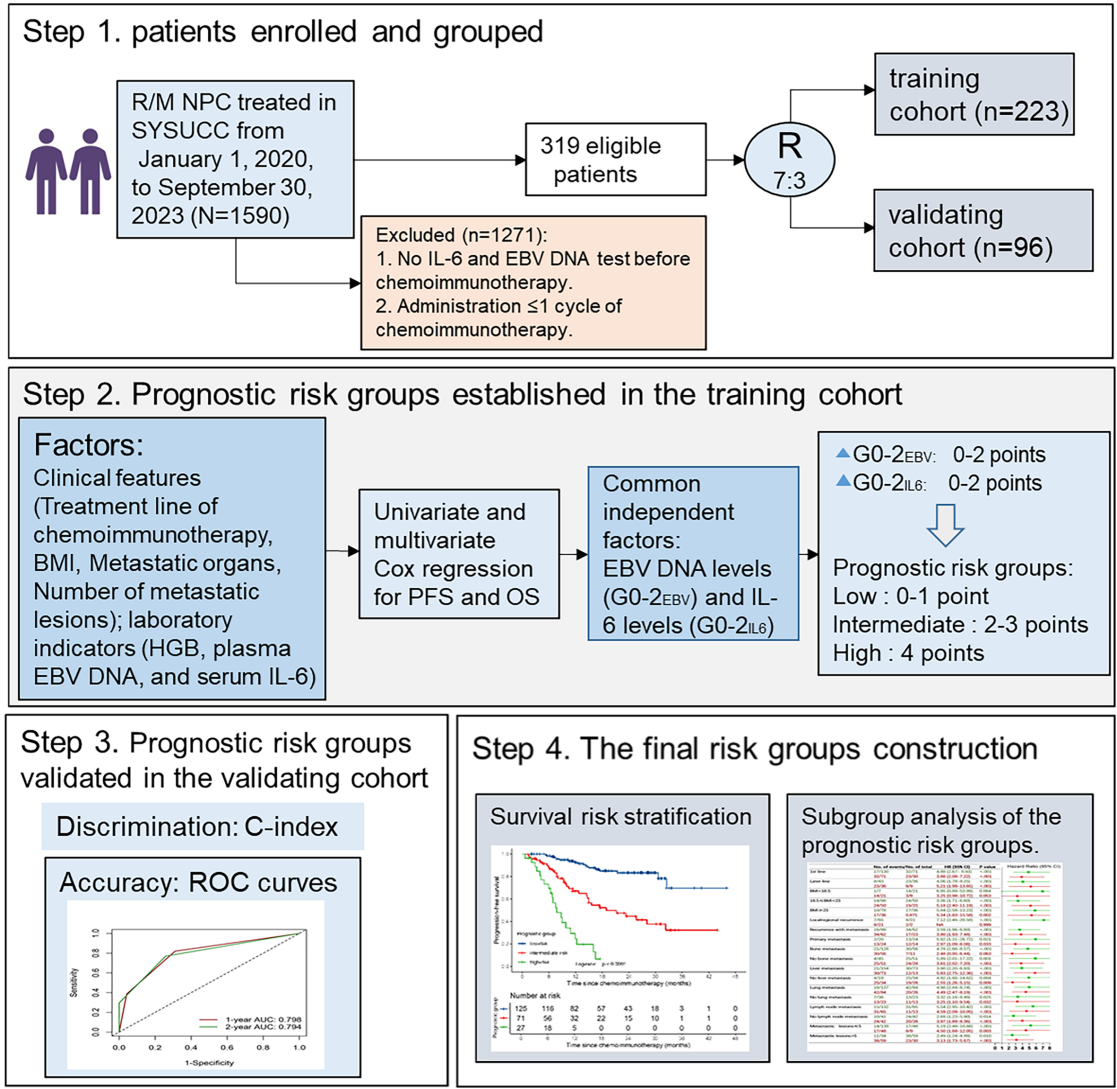
Flowchart of the study. BMI, body mass index; HB, Hemoglobin; IL-6, interleukin-6; EBV, Epstein-Barr virus; G, grade.

From January 1, 2020 to September 30, 2023, a total of 1590 patients with R/M NPC at Sun Yat-sen University Cancer Center (SYSUCC) were screened for inclusion in this study. The entry criteria were as follows: (1) patients with pathologically confirmed WHO type I, II, or III NPC; (2) patients with local or regional recurrence confirmed pathologically or distant metastasis confirmed pathologically or radiologically; (3) patients receiving palliative platinum-based chemotherapy combined with PD-1 mAb as first-line or later-line treatment; (4) patients who had completed at least two cycles of chemoimmunotherapy; (5) availability of pretreatment clinical information, laboratory data and electronic medical records. The exclusion criteria included: (1) Patients who received chemoimmunotherapy as adjuvant treatment following curative therapies; (2) patients with active infection at the time of serum IL-6 testing. (3) patients diagnosed with other malignancies. At last, 319 patients were eligible for analysis, and randomly divided into a training cohort (n=223) and an independent validation cohort (n=96) at a ratio of 7:3 (**Figure 1**).

This study was approved by the Ethics Committee of Sun Yat-sen University Cancer Center (SL-B2024-565-01) with exempting the need for informed consent as it did not involve any special interventions.

### Treatment

R/M NPC patients receiving one of the following chemotherapy regimens: GP (gemcitabine+cisplatin), TPF (docetaxel+cisplatin+5-fluorouracil), TPC (docetaxel+cisplatin+capecitabine), TP (docetaxel+cisplatin) or PF (cisplatin+5-fluorouracil), combined with PD-1 mAb (Toripalimab, Camrelizumab, Sintilimab, Tislelizumab, Pembrolizumab, or Nivolumab) for a maximum of six cycles. Maintenance therapy with PD-1 mAb was continued until disease progression, death, the occurrence of intolerable toxicities, a maximum of two years or upon the patient’s request to discontinue. Carboplatin, nedaplatin, or lobaplatin were accepted as alternatives to cisplatin (31–34). Radiotherapy or surgery therapy for locoregional or metastatic lesions was permitted during chemoimmunotherapy according to the oncologists’ decisions. Detailed information on radiotherapy was provided in **Supplementary Methods** in the **Supplementary Material**.

### Study endpoints

The endpoint of this study was PFS, defined as the time from the initiation of chemoimmunotherapy to either disease progress or death caused by any reason, censored (patients alive without progression at the last clinic date), or the last follow-up. Another endpoint was OS, defined as the time from the initiation of chemoimmunotherapy to death caused by any reason, censored, or the last follow-up visit.

### Follow-up and evaluation

During chemoimmunotherapy, patients underwent regular examinations every 2-3 cycles. For those on PD-1 mAb monotherapy, examinations were conducted every 2-4 cycles. After completing treatment, follow-up visits were scheduled at least every three months during the first two years, every six months from the third to fifth year, and annually thereafter. The last follow-up date was May 30, 2024. Regular evaluations included nasopharyngeal and cervical magnetic resonance imaging (MRI), thoracic computed tomography (CT) scans, abdominal CT scans or ultrasound, bone scans, or positron emission tomography/computed tomography (PET/CT). All radiological imaging assessments were according to the RECIST 1.1 criteria.

### Predictors

Potential predictors were pre-specified based on clinical experience and current literature regarding risk stratification for disease progression and mortality in NPC (25, 35–37), All predictors were collected at pretreatment, including sex, age, T stage, N stage, metastatic organs, number of metastatic lesions, treatment line of chemoimmunotherapy, hemoglobin, plasma EBV DNA copy number and serum IL-6 concentration. Laboratory values (including hemoglobin, plasma EBV DNA copy number, and serum IL-6 concentration) were included if obtained within one week prior to chemoimmunotherapy initiation. If multiple values were available, the value closest to the treatment start date was selected. These values were frequently, though not always, obtained on day 1 of cycle 1.

EBV DNA copy number and IL-6 concentration were tested in the laboratory of our hospital. Plasma EBV DNA copy numbers were measured using real-time quantitative polymerase chain reaction (qPCR, Sansure Biotech, Changsha, China). Amplification was performed on an Applied Biosystems 7500 sequence detector, with data analysis conducted using the sequence detection system software (version 1.6.3, Foster City, CA) developed by Applied Biosystems, with a detection limit of 0 copy/mL. Serum IL-6 concentrations was determined using commercially available human IL-6 quantitative enzyme-linked immunosorbent assay (ELISA) kits (R&D Systems, Minneapolis, MN, USA), with a detection limit of 2.5pg/mL.

### Statistical analysis

Both IL-6 concentration and EBV DNA copy number values were converted into categorical variables for analysis. Firstly, in the training cohort, IL-6 values were divided into four subgroups using integral quartile cut-off values. Then, pairwise comparisons were performed using the log-rank test to merge subgroups with similar survival outcomes. Finally, the subgroups with distinct survival differences were identified. The same method was applied to group EBV DNA values. To prevent over-optimistic results from developing and testing solely within the training set, validation was performed in the independent validation cohort.

The T-test or Mann-Whitney U test was used to compare continuous variables, while the chi-square or Fisher’s exact test was used to compare categorical variables. Cox proportional hazard models were employed to calculate hazard ratios (HRs) and 95% confidence intervals (CIs) to assess the relationship between variables and PFS/OS. Survival curves were estimated utilizing the Kaplan-Meier method and compared with the log-rank test.

The prognostic risk grouping model was established in the training cohort using the following steps: Firstly, variables P< 0.2 in the univariate Cox regression analyses were selected for further evaluation. Secondly, a backward stepwise multivariable Cox regression model was used to identify independent predictors of PFS and OS. Variables with a significance level of P < 0.05 in the multivariate analyses were retained. Thirdly, a prognostic score was constructed based on the significant and common predictors identified for PFS and OS. Finally, subgroups with similar survival curves were merged to create the final prognostic risk grouping model. The model was evaluated in two aspects: (1) discrimination was measured by the C-index and its 95% CI; (2) accuracy was evaluated by time-dependent ROC curves and area under the curve (AUC) for PFS and OS at 12 and 24 months.

The prognostic risk grouping model was confirmed in the validation set and across different subgroups of the full set, including first and later lines, primary and recurrent metastasis, different body mass indexes (BMI), bone metastasis or not, liver metastasis or not, lung metastasis nor not lymph node metastasis or not or different metastatic lesions.

All statistical analyses were performed using R studio 4.3.2 (Boston, MA, USA), GraphPad Prism 8 (GraphPad Software, Inc., San Diego, CA) and IBM SPSS Statistics 26 for Windows (SPSS Inc, Chicago, IL). Statistical significance thresholds were set at a two-tailed P value of <0.05.

## Results

### Patient characteristics (the whole cohort)

In the whole cohort of 319 patients, the median age were 49.0 years (interquartile range [IQR] 40.0-57.0), and 231 (72.41%) were treated with chemotherapy plus PD-1 mAb as first-line therapy. 58 patients (18.18%) had *de novo* metastatic NPC, while 173 patients (54.23%) experienced both local and distant recurrence. The median serum concentration was 7.51 pg/mL (IQR 3.00-18.39) for IL-6 and 996.00 copies/mL (IQR 64.20-13911.00) for EBV DNA.

The median follow-up time of the full set was 18.5 months (IQR 8.9-25.1). During the study period, 111 patients (34.80%) experienced disease progression, and 69 patients (21.63%) died. The pooled analysis of 319 patients revealed a median PFS (mPFS) of 28.10 months (95% CI 24.2-33.5), while the median OS (mOS) was not reached. As shown in **Table 1**, the pre-treatment clinical characteristics and laboratory results of the training and validating cohorts were balanced.

**Table 1:** Characteristics of patients in 319 R/M NPC patients.

	Total (n = 319)	training cohort (n = 223)	validation cohort (n = 96)	P value*
Age (years)				
median (IQR)	49.00 (40.00-57.00)	47.00 (39.00-56.00)	50.00 (42.00-58.00)	0.179
Sex				
male	245 (76.80)	165 (73.99)	80 (83.33)	0.070
female	74 (23.20)	58 (26.01)	16 (16.67)	
BMI, kg/m^2^				
median (IQR)	22.00 (20.00-24.10)	21.80 (19.87-24.00)	22.52 (20.60-24.40)	0.169
Hemoglobin concentration (continuous, g/dL)				
mean ± SD	12.56 ± 2.18	12.47 ± 2.05	12.77 ± 2.44	0.254
IL-6 concentration (pg/mL)				
median (IQR)	7.51 (3.00-18.39)	7.02 (3.11-18.39)	8.42 (2.72-17.81)	0.835
EBV-DNA copy number (copies/mL)				
median (IQR)	996.00 (64.20-13911.00)	1010.00 (66.00-14800.00)	949.50 (64.05-12230.75)	0.833
Treatment line(s) of chemoimmunotherapy†				0.891
1	231 (72.41)	161 (72.20)	70 (72.92)	
≥ 2	88 (27.59)	62 (27.80)	26 (27.08)	
Chemotherapy regime				0.503
GP	152 (47.65)	109 (48.88)	43 (44.79)	
TP	56 (17.55)	42 (18.83)	15 (15.63)	
TPF/TPC	62 (19.44)	40 (17.94)	22 (22.92)	
PF	49 (15.36)	32 (14.35)	17 (17.71)	
Disease status				0.854
primary metastasis	58 (18.18)	41 (18.39)	17 (17.71)	
local-regional recurrence only	88 (27.59)	63 (28.25)	25 (26.04)	
recurrence with metastasis	173 (54.23)	121 (54.26)	52 (54.17)	
T stage ^§^				0.879
(r)T0-3	271 (84.95)	189 (84.75)	82 (85.42)	
(r)T4	48 (15.05)	34 (15.25)	14 (14.58)	
N stage ^§^				0.320
(r)N0-2	255 (79.94)	175 (78.48)	80 (83.33)	
(r)N3	64 (20.06)	48 (21.52)	16 (16.67)	
Bone metastases				0.864
no	195 (61.13)	137 (61.43)	58 (60.42)	
yes	124 (38.87)	86 (38.57)	38 (39.58)	
Liver metastases				0.362
no	240 (75.24)	171 (76.68)	69 (71.88)	
yes	79 (24.76)	52 (23.32)	27 (28.12)	
Lung metastases				0.626
no	247 (77.43)	171 (76.68)	76 (79.17)	
yes	72 (22.57)	52 (23.32)	20 (20.83)	
Distant lymph node(s) metastases				0.471
no	210 (65.83)	144 (64.57)	66 (68.75)	
yes	109 (34.17)	79 (35.43)	30 (31.25)	
Number of metastasis lesions				0.997
≤ 5	196 (61.44)	137 (61.43)	59 (61.46)	
> 5	123 (38.56)	86 (38.57)	37 (38.54)	
Disease progression				0.506
no	208 (65.20)	148 (66.37)	60 (62.50)	
yes	111 (34.80)	75 (33.63)	36 (37.50)	
Death				0.508
no	250 (78.37)	177 (79.37)	73 (76.04)	
yes	69 (21.63)	46 (20.63)	23 (23.96)	

^*^P values were calculated using the t-test or Mann-Whitney U test for continuous variables, and the χ2 test or Fisher exact for categorical variables.

^§^UICC/AJCC 8^th^ stage system.

†Systemic treatment (chemotherapy with or without antiangiogenic drugs) after diagnosing recurrent or metastatic NPC.

SD, standard deviation; BMI, body mass index; GP, gemcitabine plus cisplatin; TP: docetaxel and platinum; TPF/TPC: docetaxel, platinum and 5-fluorouracil/docetaxel, platinum and capecitabine; PF, platinum and 5-fluorouracil; IQR, interquartile range; IL-6, interleukin-6; EBV, Epstein-Barr virus; (r), recurrence.

### Determination of different grades for IL-6 and EBV-DNA values (training cohort)

**Figure 2:**
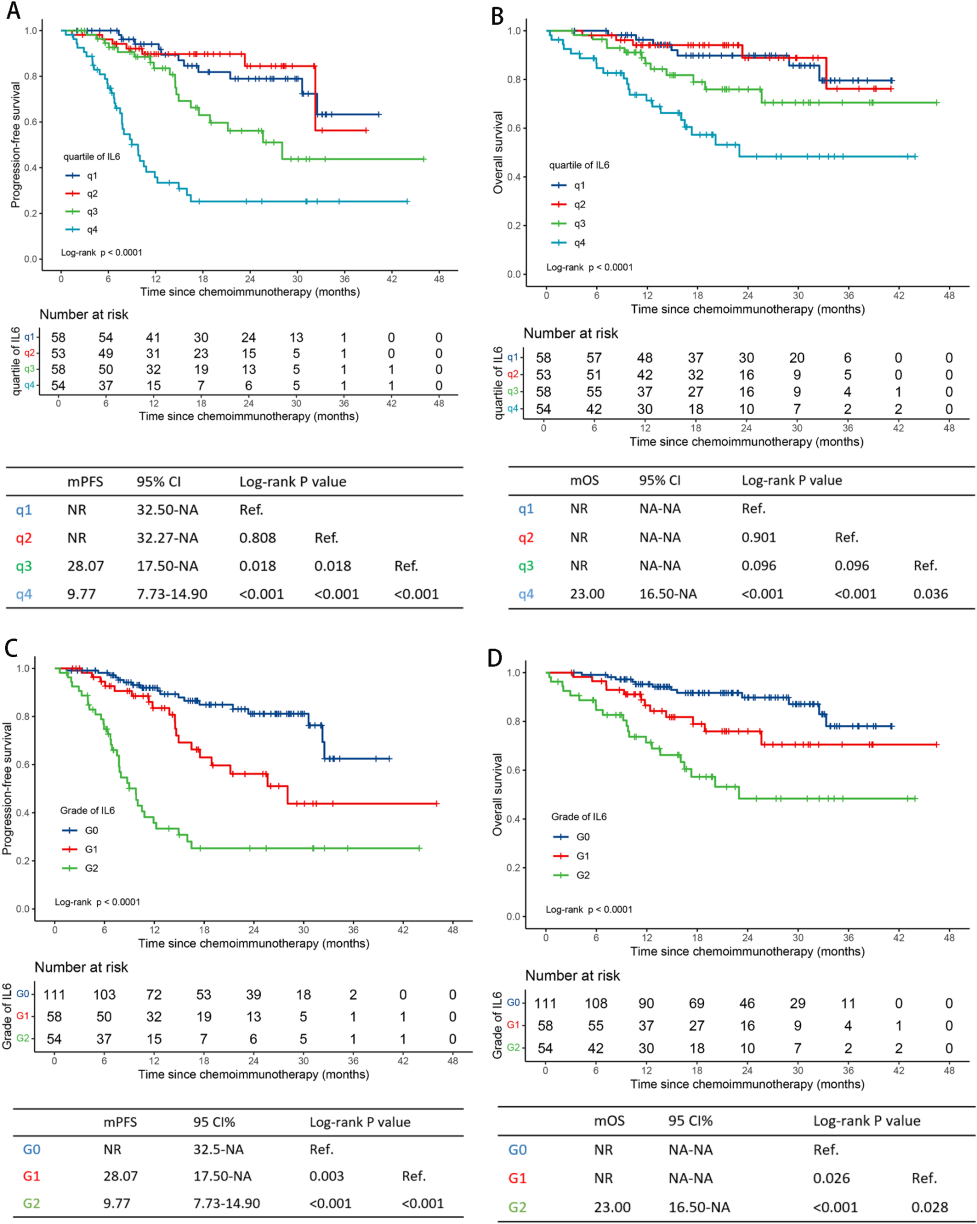
Survival curves by serum IL-6 levels. Progression-free survival curves for q1-4_IL6_ levels and G0-2_IL6_ levels **(A, C)**. Overall survival curves for q1-4_IL6_ levels and G0-2_IL6_ levels **(B, D)** in the training cohort. IL-6, interleukin-6; q, quartile; G, grade; NR, not reached; NA, not applicable.

The integral quartile ranges of IL-6 values were as follows: <3.0 (q1), 3.0-7.0 (q2), 7.0-18.0 (q3), and >18.0 (q4) pg/mL (**Supplementary Table S1**). No significant differences in PFS or OS were observed between subgroups q1 and q2 (**Figures 2A, B**). Therefore, we combined subgroups q1 and q2 into a single group, G0_IL6_. Three distinct survival subgroups of IL-6 were identified, representing three different survival risk grades (G0-2_IL6_), as shown in **Figures 2C, D**.

Similarly to EBV DNA value grouping, three different survival risk groups (G0-2_EBV_) were formed after merging subgroups q1 and q2. The cut-off values were 1000 and 15000 copies/ml (**Supplementary Table S1**, **Supplementary Figures S2A–D**).

### Establishment of the prognostic risk grouping (training cohort)

In the training cohort, the number of metastatic lesions (>5 VS. ≤5, HR =2.09 [95% CI 1.23-3.55], P=0.006), pretreatment serum IL-6 levels (G1_IL6_ VS. G0_IL6_; HR=1.98 [1.05-3.72], P=0.034; G2_IL6_ VS. G0_IL6_, HR=4.80 [2.54-9.06], P < 0.001) and EBV DNA levels (G1_EBV_ VS. G0_EBV_, HR=1.72 [0.91-3.28], P=0.097; G2_EBV_ VS. G0_EBV_, HR=2.92 [1.57-5.43], P < 0.001) were identified as independent prognostic factors for PFS in multivariable analyses. For OS, EBV DNA (G1_EBV_ VS. G0_EBV_, HR=2.16 [0.89-5.21], P=0.087; G2_EBV_ VS. G0_EBV_, HR=5.82 [2.56-13.25], P < 0.001) and IL-6 levels (G1_IL6_ VS. G0_IL6_, HR=1.52 [0.68-3.40], P=0.311; G2_IL6_ VS. G0_IL6_, HR=2.47 [1.17-5.20], P =0.017) were identified as independent prognostic factors (**Table 2**).

**Table 2:** Multivariate Cox regression analyses of prognostic factors for PFS and OS in the training cohort.

Variables	PFS		OS	
	HR (95% CI)	P value	HR (95% CI)	P value
BMI (kg/m2)				
< 18.5	1.00 (Reference)		1.00 (Reference)	
18.5–22.9	0.93 (0.47 - 1.86)	0.843	1.05 (0.44 - 2.48)	0.911
≥ 23	0.93 (0.42 – 2.11)	0.877	0.97 (0.35 - 2.70)	0.960
T stage^§^				
(r)T0-3	–		1.00 (Reference)	
(r)T4	–		1.77 (0.91 - 3.42)	0.467
N stage^§^				
(r)N0-2	1.00 (Reference)		–	
(r)N3	1.50 (0.84 - 2.71)	0.173	–	
Bone metastases				
no	1.00 (Reference)		1.00 (Reference)	
yes	0.71 (0.84 - 2.71)	0.221	1.13 (0.57 - 2.24)	0.723
Liver metastases				
no	1.00 (Reference)		1.00 (Reference)	
yes	1.30 (0.69 - 2.42)	0.418	1.27 (0.61 - 2.61)	0.521
Lung metastases				
no	1.00 (Reference)		–	
yes	0.95 (0.50 - 1.81)	0.876	–	
Distant lymph node metastases				
no	1.00 (Reference)		1.00 (Reference)	
yes	0.87 (0.47 - 1.67)	0.707	1.00 (0.46 - 2.19)	0.985
Number of metastasis lesions				
0-5	1.00 (Reference)		1.00 (Reference)	
> 5	2.09 (1.23-3.55)	**0.006**	1.67 (0.74 - 3.78)	0.217
Treatment line(s) of chemoimmunotherapy^†^				
1	1.00 (Reference)		–	
≥2	1.18 (0.68 - 2.08)	0.550	–	
Hemoglobin				
	0.93 (0.82 – 1.06)	0.292	0.95 (0.83 - 1.07)	0.392
IL-6 levels				
G0_IL6_	1.00 (Reference)		1.00 (Reference)	
G1_IL6_	1.98 (1.05-3.72)	**0.034**	1.52 (0.68-3.40)	0.311
G2_IL6_	4.80 (2.54-9.06)	**<.001**	2.47 (1.17-5.20)	**0.017**
EBV DNA levels				
G0_EBV_	1.00 (Reference)		1.00 (Reference)	
G1_EBV_	1.72 (0.91-3.28)	0.097	2.16 (0.89-5.21)	0.087
G2_EBV_	2.92 (1.57-5.43)	**<.001**	5.82 (2.56-13.25)	**<.001**

^§^UICC/AJCC 8^th^ stage system.

†Systemic treatment (chemotherapy with or without antiangiogenic drugs) after diagnosing recurrent or metastatic NPC.

HR, hazard ratio; PFS, progression-free survival; OS, overall survival; IQR, interquartile range; BMI, body mass index; IL6, interleukin-6; EBV, Epstein-Barr virus; (r), recurrence; G, grade.

The bold values indicate P values with statistically significant difference.

**Figure 3:**
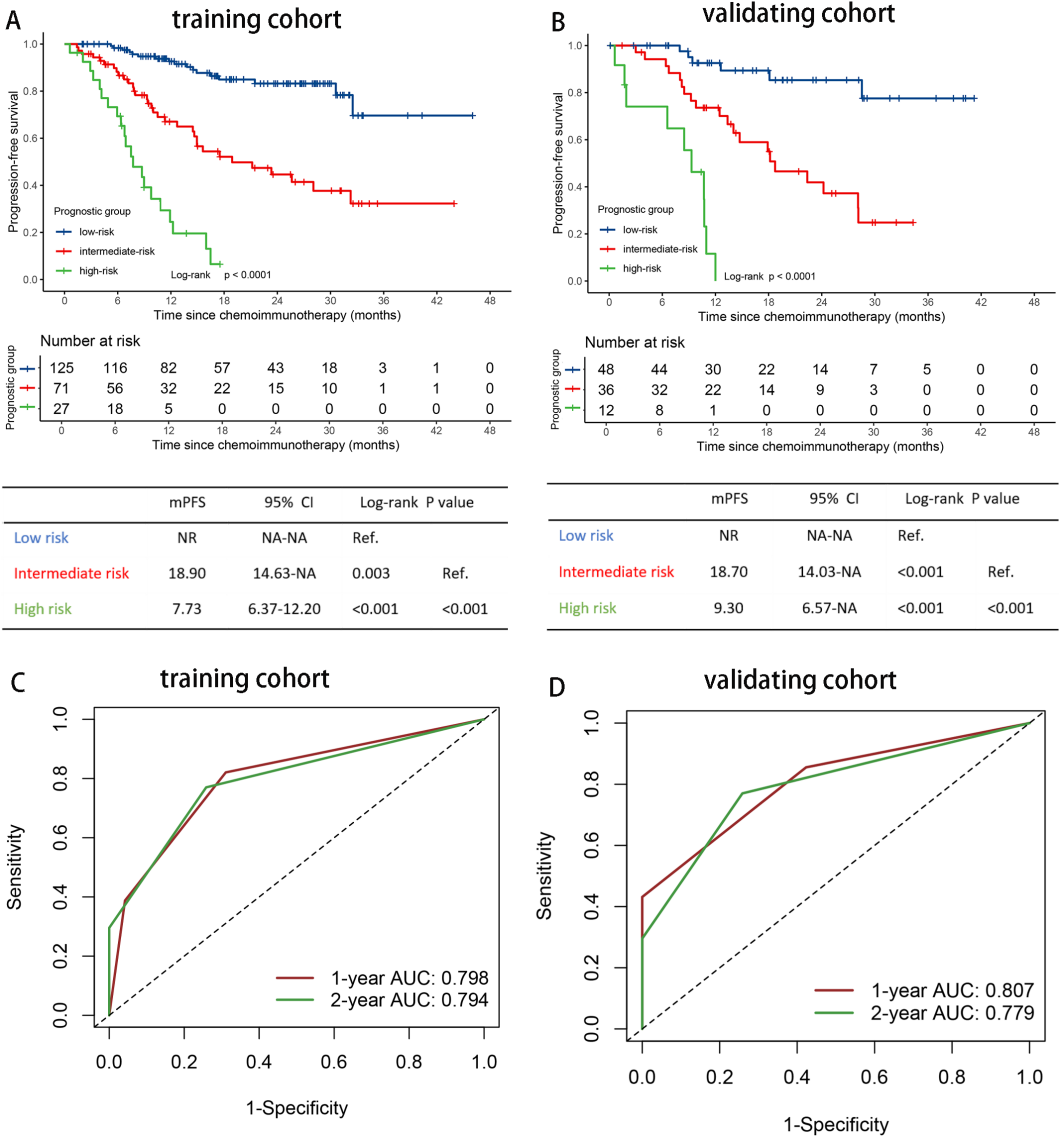
Risk stratification and model evaluation. Prognostic risk stratification and model evaluation. Kaplan Meier curves of PFS for patients stratified by prognostic risk groups in **(A)** the training and **(B)** the validation cohort. The time-dependent ROC curves of the prognostic risk grouping for predicting the 1-, and 2-year PFS rate in **(C)** the training and **(D)** the validation cohort. ROC, receiver operating characteristic curves; AUC, area under the curve; HR, hazard ratio; PFS, progression-free survival; IL-6, interleukin-6; NR, not reached; NA, not applicable.

As both EBV DNA and IL-6 levels were prognostic factors independently for PFS and OS, we developed a simple prognostic score based on these two variables. Given that the prognostic HRs of G_EBV_0-2 or G_IL6_0-2 approximately doubled with the increasing risk grades in multivariable survival analysis, we assigned 0-2 points for G_EBV_0-2 and G_IL6_0-2 separately. This resulted in a prognostic score ranging from 0 to 4 points. Pairwise comparisons using the log-rank test revealed no significant difference in survival outcomes between subgroups with 0 and 1 point, or between subgroups with 2 and 3 points (**Supplementary Figures S2A, B**). Therefore, we combined 0 and 1 point subgroups as the low-risk group, subgroups 2 and 3 points as the intermediate-risk group. The final prognostic risk grouping model consisted of three categories: low risk (0-1 point), intermediate risk (2-3 points), and high risk (4 points) (**Figure 3A**, **Supplementary Figure S3A**).

### Evaluation of the prognostic risk grouping (training cohort)

The C-indexes were 0.753 (95% CI 0.700-0.806) for PFS, and 0.740 (95% CI 0.667-0.813) for OS in the training set. Using time-dependent AUCs, PFS discrimination was 0.798 (95% CI 0.724-0.872) at 12 months and 0.794 (95% CI 0.722-0.866) at 24 months (**Figure 3C**). For OS, the discrimination were 0.732 (95% CI 0.620-0.819) at 12 months and 0.827 (95% CI 0.748-0.908) at 24 months (**Supplementary Figure S3C**).

Patients with 0-1 point (low risk, n=125) had the longest median PFS and OS (PFS: NR; OS: NR), followed by those with 2-3 points (intermediate risk, n=71) (PFS, 18.90 months [95%CI 9.30-28.50]; OS, NR), and those with 4 points (high risk, n=27) had the poorest outcomes (PFS, 7.73 months [95% CI 4.94-10.53]; 13.6 months [95% CI 5.49-21.71]) (**Figure 3A**, **Supplementary Figure S3A**). The overall P value of PFS and OS were <0.001.

### Validation of the prognostic risk grouping (validation cohort)

In the validating set, the C-indexes of the prognostic risk grouping model were 0.757 (95% CI 0.682-0.832) for PFS and 0.766 (95% CI 0.683-0.850) for OS. For the time-dependent AUCs, PFS discrimination was 0.807 (95% CI 0.700-0.916) at 12 months and 0.779 (95% CI 0.672-0.886) at 24 months (**Figure 3D**). For OS, the AUCs were 0.787 (95% CI 0.672-0.902) at 12 months and 0.782 (95% CI 0.667-0.897) at 24 months (**Supplementary Figure S3D**).

The median PFS of patients with 0-1 point (low risk, n=48) was not reached, while for those with 2-3 points (intermediate risk, n=36), the median PFS was 18.73 months (95%CI 12.39-25.08). For patients with 4 points (high risk, n=12), the median PFS was 9.30 months (95%CI 6.14-12.46) (P < 0.001) (**Figure 3B**). The median OS for patients in the high-risk group (4 points) was 17.93 months (95% CI 0.00-37.96), while the median OS for the low- and intermediate-risk groups were not reached (P < 0.001) (**Supplementary Figure S3B**).

### The prognostic risk grouping in different subgroups (the whole cohort)

In the whole set, the median PFS was not reached for the low-risk group (n = 173), 18.73 months (95% CI 11.87-25.60) for the intermediate-risk group (n = 107), and 8.73 months (95% CI 6.68-10.79) for the high-risk group (n = 39). The median OS was 16.47 months (95% CI 8.40-24.53) for the high-risk group, which was not reached for the low- and intermediate-risk groups. To assess the sensitivity of the prognostic risk grouping model, we applied it to different subgroups in the entire cohort (n=319).

**Figure 4:**
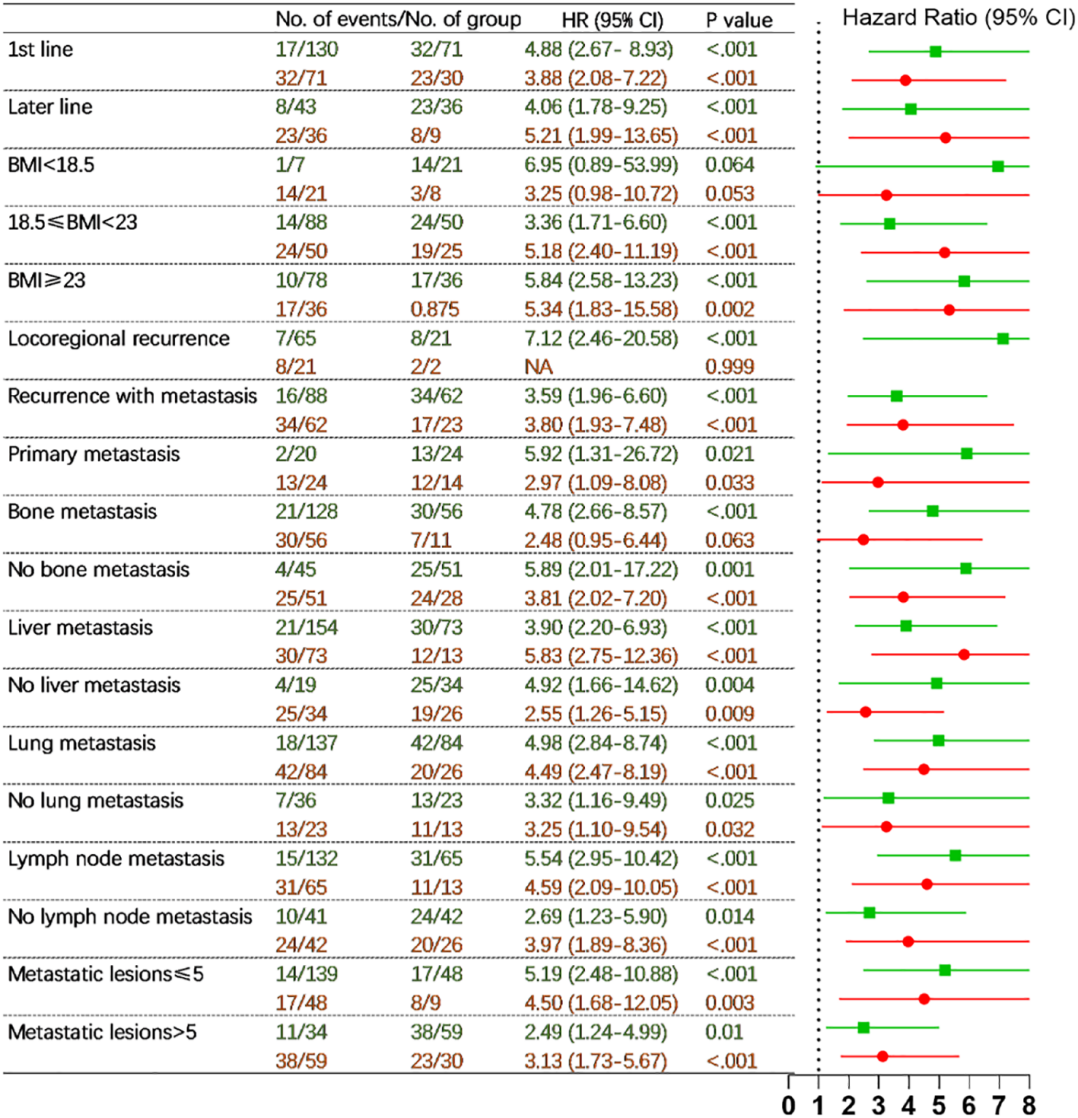
Hazard ratios for disease progression comparing three prognostic risk groups (low- vs. intermediate-risk in green and intermediate- vs. high-risk in red) in different subgroups in the full cohort (adjusting sex, age, hemoglobin, and chemotherapy regimens). HR, hazard ratio; CI, confidence interval; BMI, body mass index; NA, not applicable. P values indicated the levels of statistical differences.

In patients who received chemoimmunotherapy as first-line, median PFS was not reached for the low-risk group (n = 130), 25.03 months (95% CI 15.93-34.13) for the intermediate-risk group (n = 71), and 13.60 months (95% CI 8.65-18.55) for the high-risk group (n = 30). In patients who underwent immunotherapy in the later line, median PFS was not reached for the low-risk group (n = 43), 23.33 months (95% CI 14.34-32.32) for the intermediate-risk group (n = 36), and 8.47 months (95% CI 3.50-13.43) for the high-risk group (n = 9) (p <0.001) (**Figure 4**). A similar trend was observed for OS (**Supplementary Figure S4**).

Comparable results were also obtained in further subgroups including patients with BMI <18.5, 18.5-23 and ≥ 23 kg/m^2^, locoregional recurrence, recurrence with metastasis and primary metastasis, absence and presence of bone metastasis, absence and presence of liver metastasis, absence and presence of lung metastasis, absence and presence of lymph node metastasis, metastatic lesions ≤ 5 and >5 (**Figure 4**, **Supplementary Figure S4**).

## Discussion

In our study, pretreatment IL-6 levels were identified as an independent prognostic factor for survival in R/M NPC patients receiving chemoimmunotherapy. And we combined pretreatment serum IL-6 and plasma EBV DNA levels to construct a simple but applicable prognostic risk grouping model. This model effectively predicted the likelihood of improved PFS and OS in R/M NPC patients treated with chemoimmunotherapy. Specifically, patients with pretreatment serum IL-6 concentrations exceeding 18.0 pg/ml and EBV DNA copy numbers exceeding 15,000 copies/ml had poorer survival outcomes compared to those who met neither of these criteria. Results were highly consistent in an independent validation and various subgroups. Importantly, our prognostic risk grouping model demonstrated a strong association not only with PFS but also with OS for R/M NPC patients undergoing chemoimmunotherapy.

There is a strong rationale for combining IL-6 and EBV DNA to predict survival outcomes in R/M NPC patients undergoing chemoimmunotherapy. The secretion of IL-6 was stimulated by inflammation or cancer processes (38). Inflammation, regarded as a hallmark of cancer, contributed to tumorigenesis and cancer progression (39), closely linking IL-6 to the mechanisms underlying cancer development and advancement. Furthermore, IL-6 played an critical role in regulating B cell and T cell responses and coordinating the activities of the innate and adaptive immune systems (38), suggesting its association with both the efficacy and toxicity of immunotherapy (25, 26, 38, 40).

High level of IL-6 was correlative with tumor proliferation, poorer chemoimmunotherapy efficacy and worse survival outcomes (26, 28, 29). As presented in **Supplementary Table S1**, patients with IL-6 levels of 18pg/mL or higher had the poorest median PFS (10.40 months) and OS (20.10 months). Conversely, patients with IL-6 levels of 7pg/mL or lower had the longest median PFS (33.60 months) and a not-reached median OS. Higher serum IL-6 levels were also significantly associated with aggressive disease behaviors, such as M1 stage, multi-metastases, and liver metastases (**Supplementary Table S3**).

IL-6 has been shown to potentiate several essential transcriptional molecules involved in cell proliferation, including G1/S- specific cyclin D1, the proto-oncogene MYC, BCL-XL, and the apoptosis inhibitor surviving. IL-6 also promoted angiogenesis, tumor invasion, and metastasis through the regulation of hypoxia-inducible factor 1α (HIF-1α), matrix metalloproteinases (MMP2, MMP7, MMP9), and vascular endothelial growth factor (VEGF) (41). Moreover, IL-6 facilitates the metastatic spread of invasive cancer cells by inducing transcriptional activators of epithelial-mesenchymal transition (41). Elevated IL-6 levels also inhibited the production of CD8+ and CD4+ T lymphocyte cells, creating a “cold” tumor microenvironment (TME) that was unfavorable for anti-PD-1 therapy (40). Besides, IL-6 promoted the recruitment of myeloid-derived suppressor cells (MDSCs), which further inhibited the antitumor effectiveness of active T cells (42). Meanwhile, IL-6 induced chemotherapeutic resistance by activating autophagy via IL-6/JAK2/BECN1 signaling pathway, as seen in colorectal cancer (43).

EBV DNA levels were widely recognized as a robust predictor of treatment efficacy and long-term prognosis in NPC, showing correlations with progression-free and recurrence-free survival (15, 18, 20, 44, 45). Our results were consistent with these established findings. Prospective clinical studies have demonstrated that pre-treatment EBV-DNA levels exceeding 2000 or 4000 copies/ml were indicative of higher risks of metastasis and mortality in patients with locally advanced NPC (46, 47). Interestingly, it was observed that EBV-positive NPC cells would contribute to treatment resistance by inducing cancer-associated fibroblast-mediated immunosuppression through YAP1/FAPα signaling (48).

Together, these findings suggested that both IL-6 and EBV DNA levels influence tumor cells directly or indirectly by promoting an immunosuppressive milieu and treatment resistance, which ultimately hampered the efficacy of chemoimmunotherapy. Based on these insights, we integrated IL-6 and EBV DNA levels to develop a prognostic risk stratification model for R/M NPC patients treated with chemoimmunotherapy, with the aim to predict patient prognosis and optimize therapeutic strategies in clinical practice.

This study had several limitations. As a retrospective analysis, the lack of scheduled radiological assessments might affect the evaluation of PFS. Furthermore, the sample size was limited, however, we reviewed all patients consecutively to minimize selection bias. Additionally, 35.5% of patients received chemoimmunotherapy as a second-line or later treatment rather than a first-line option, thus we analyzed the model’s sensitivity across first- and later-line subgroups. Additionally, mOS could not be determined due to the insufficient follow-up duration, so we used PFS as an alternative endpoint and focused on survival outcomes at 12 and 24 months. Last but not least, an external validation was absent because of a rare detection of pretreatment IL-6 value and we can’t collect enough external data.

In conclusion, our prognostic risk grouping model was simple but associated with the survival outcomes of both PFS and OS in patients with R/M NPC receiving chemoimmunotherapy. This model could assist clinicians in making an initial prognostic assessments. However, a prospective, large-scale, long-term, randomized controlled clinical trial is warranted to validate this prognostic risk stratification model and explore the potential benefits of anti-IL6 therapies.

## Data Availability

The datasets presented in this study can be found in online repositories. The names of the repository/repositories and accession number(s) can be found in the article/**Supplementary Material**.
