# Supplementary material for: Combining interleukin 6 and EBV DNA levels predicts survival outcomes for patients with recurrent or metastatic nasopharyngeal carcinoma receiving chemoimmunotherapy
Source: Front Immunol. 2025 Mar 14;16:1560897. doi: 10.3389/fimmu.2025.1560897 (PMC11949991; doi:10.3389/fimmu.2025.1560897)
Supplement: Supplementary file 1 [file Table1.docx]

**Supplementary Materials for**

**Combining Interleukin 6 and EBV DNA levels predicts survival outcomes for patients with recurrent or metastatic nasopharyngeal carcinoma receiving chemoimmunotherapy**

1. Supplementary Methods
2. Supplementary Tables:

Table S1. Grading of serum IL-6 and EBV DNA according to survival (training cohort).

Table S2. Univariate Cox regression analyses of prognostic factors for PFS and OS in the training cohort.

Table S3. Correlation between G0-2_IL-6_ levels and clinical parameters (the full cohort).

1. Supplementary Figures:

Figure S1. Survival curves by pretreatment plasma EBV DNA levels.

Figure S2. Prognostic scores based on IL-6 and EBV DNA levels.

Figure S3. Prognostic risk stratification and model evaluation.

Figure S4. Hazard ratios for death comparing three prognostic risk groups (low- vs. intermediate-risk in green and intermediate- vs. high-risk in red) in different subgroups of the full cohort (adjusting sex, age, hemoglobin, and chemotherapy regimens).

Supplementary Methods:

The chemotherapy regimens included: 1) GP (1000 mg/m^2^ gemcitabine administered intravenously on days 1 and 8 plus platinum), 2) TP (75 mg/m^2^ docetaxel administered intravenously on day 1 plus platinum), 3) PF (platinum and 750–1000 mg/m^2^ 5-fluorouracil civ96-120h), 4) TPF (60 mg/m^2^ docetaxel administered intravenously on day 1, platinum, and 500–800 mg/m^2^ 5-fluorouracil civ120h), and 5) TPC (60 mg/m^2^ docetaxel administered intravenously on day 1, platinum, and 1000 mg/m^2^ oral capecitabine twice daily on days 1–14). Platinum included cisplatin (80–100 mg/m^2^ administered intravenously on day 1 or a total of 80–100 mg/m^2^ administered intravenously on days 1–3), and nedaplatin (80–100 mg/m^2^ administered intravenously on day 1).

The anti-PD-1 mAbs regimens were: 1) sintilimab (200 mg administered intravenously on day 1), 2) camrelizumab (200 mg administered intravenously on day 1), 3) toripalimab (240 mg administered intravenously on day 1), 4) tislelizumab (200 mg administered intravenously on day 1), 5) pembrolizumab (200 mg administered intravenously on day 1), 6) nivolumab (200 mg administered intravenously on day 1).

Surgery therapy included: cervical lymph node dissection, resection for lung metastases, ablation for liver or lung metastases.

For de novo metastatic NPC patients receiving LRRT, the doses for the primary site and neck lymph node were 60–70Gy/30–35 fractions for the primary gross tumor volume (GTV), 60–66Gy/30–35 fractions for the clinical target volume (CTV)-1, and 54–56Gy/30–35 fractions for CTV-2. For locoregionally recurrent NPC patients receiving reirradiation, the prescription dose was 50–60Gy/25–30 fractions.

RT for metastatic lesions, including those in the liver, bones, lungs, and distant lymph nodes. Patients received a GTV dose of 45Gy/15 fractions using the IMRT technique or a GTV dose of 27–40Gy/3–8 fractions to the liver metastases using the stereotactic body radiotherapy (SBRT) technique. For bone metastases, a GTV dose was 40–66Gy/20–33 fractions using the IMRT technique or a 30-40Gy/5-8 fractions dose. To the lungs metastases, patients received 40-54Gy/5-9 fractions via SBRT and 50Gy/25 fractions via IMRT, For the axillary metastatic lymph nodes, patient received 30-49Gy/5-7 fractions by SBRT and 50-66Gy/25-33 fractions by IMRT.

**Table S1. Grading of serum IL-6 and EBV DNA according to survival (training cohort).**

| **variables** | **groups** | **percentile** | **ranges*** | **PFS**  **HR ( 95% CI) P value** | | **OS**  **HR ( 95% CI) P value** | | **Grading** | **PFS**  **HR ( 95% CI) P value** | | **OS**  **HR ( 95% CI) P value** | |
| --- | --- | --- | --- | --- | --- | --- | --- | --- | --- | --- | --- | --- |
| IL-6 | q1_IL6_ | P0-P25 | <3 | 1 (reference) | - | 1 (reference) | - | G0_IL6_ | 1 (reference) | - | 1 (reference) | - |
|  | q2_IL6_ | P25-P50 | 3-7 | 1.23  (0.46 - 3.31) | 0.678 | 2.35  (0.46 - 12.13) | 0.306 |  |  |  |  |  |
|  | q3_IL6_ | P50-P75 | 7-18 | 2.64  (1.10 - 6.32) | 0.029 | 6.49  (1.46 - 28.76) | 0.014 | G1_IL6_ | 2.36  (1.20 - 4.63) | 0.013 | 3.82  (1.52 - 9.58) | 0.004 |
|  | q4_IL6_ | P75-P100 | ≥18 | 7.61  (3.36 - 17.23) | <.001 | 14.53  (3.41 - 61.96) | <.001 | G2_IL6_ | 6.80  (3.74 - 12.36) | <.001 | 8.56  (3.64 - 20.10) | <.001 |
| EBV DNA | q1_EBV_ | P0-P25 | 0-60 | 1 (reference) | - | 1 (reference) | - | G0_EBV_ | 1 (reference) | - | 1 (reference) | - |
|  | q2_EBV_ | P25-P50 | 60-1000 | 0.44  (0.20 - 0.99) | 0.050 | 0.90  (0.23 - 3.61) | 0.884 |  |  |  |  |  |
|  | q3_EBV_ | P50-P75 | 1000-15000 | 1.45  (0.74 - 2.86) | 0.244 | 2.73  (0.87 - 8.59) | 0.085 | G1_EBV_ | 2.24  (1.20 - 4.18) | 0.011 | 3.06  (1.19 - 7.91) | 0.021 |
|  | q4_EBV_ | P75-P100 | ≥15000 | 3.30  (1.74 - 6.25) | <.001 | 8.76  (3.03 - 25.32) | <.001 | G2_EBV_ | 5.13  (2.87 - 9.19) | <.001 | 10.59  (4.54 - 24.71) | <.001 |

* integering ranges from the quartile points.

Note: q1: ≥ 0th percentile and < 25percentile; q2: ≥ 25th percentile and <50th percentile; q3: ≥ 50th percentile and < 75th percentile; q4: ≥ 75th percentile.

Abbreviations: q, quartile; G, grade; P, percentile; IL-6, interleukin-6; EBV, Epstein-Barr virus.

**Table S2. Univariate Cox regression analyses of prognostic factors for PFS and OS in the training cohort.**

| **Variables** | **PFS** | | **OS** | |
| --- | --- | --- | --- | --- |
|  | **HR ( 95% CI)** | **P value** | **HR ( 95% CI)** | **P value** |
| Sex |  |  |  |  |
| male | 1.00 (Reference) |  | 1.00 (Reference) |  |
| female | 0.98 (0.57 - 1.67) | 0.943 | 0.95 (0.48 - 1.88) | 0.890 |
| Age (years) |  |  |  |  |
| < 49 | 1.00 (Reference) |  | 1.00 (Reference) |  |
| ≥ 49 | 0.91 (0.57 - 1.45) | 0.694 | 1.00 (0.56 - 1.79) | 0.994 |
| BMI (kg/m^2^) |  |  |  |  |
| < 18.5 | 1.00 (Reference) |  | 1.00 (Reference) |  |
| 18.5–22.9 | 0.58 (0.31 - 1.06) | 0.075 | 0.63 (0.28 - 1.41) | 0.262 |
| ≥ 23 | 0.36 (0.18 - 0.72) | **0.004** | 0.44 (0.18 - 1.06) | 0.068 |
| T stage^§^ |  |  |  |  |
| (r)T0-3 | 1.00 (Reference) |  | 1.00 (Reference) |  |
| (r)T4 | 1.63 (0.95 - 2.81) | 0.078 | 1.77 (0.91 - 3.42) | 0.090 |
| N stage^§^ |  |  |  |  |
| (r)N0-2 | 1.00 (Reference) |  | 1.00 (Reference) |  |
| (r)N3 | 1.67 (1.01 - 2.77) | **0.048** | 1.22 (0.62 - 2.40) | 0.571 |
| Bone metastases |  |  |  |  |
| no | 1.00 (Reference) |  | 1.00 (Reference) |  |
| yes | 1.38 (0.87 - 2.17) | 0.166 | 2.22 (1.24 - 3.98) | **0.007** |
| Liver metastases |  |  |  |  |
| no | 1.00 (Reference) |  | 1.00 (Reference) |  |
| yes | 3.15 (1.98 - 5.01) | **<.001** | 3.36 (1.86 - 6.07) | **<.001** |
| Lung metastases |  |  |  |  |
| no | 1.00 (Reference) |  | 1.00 (Reference) |  |
| yes | 1.77 (1.10 - 2.87) | **0.020** | 1.04 (0.53 - 2.05) | 0.904 |
| Distant lymph node metastases | | | | |
| no | 1.00 (Reference) |  | 1.00 (Reference) |  |
| yes | 2.57 (1.63 - 4.05) | **<.001** | 2.55 (1.43 - 4.55) | **0.002** |
| Number of metastasis lesions | | | | |
| 0-5 | 1.00 (Reference) |  | 1.00 (Reference) |  |
| > 5 | 3.84 (2.39 - 6.20) | **<.001** | 3.51 (1.93 - 6.40) | **<.001** |
| Treatment line(s) of chemoimmunotherapy^⁺^ | | | | |
| 1 | 1.00 (Reference) |  | 1.00 (Reference) |  |
| ≥2 | 1.40 (0.87 - 2.23) | 0.164 | 1.24 (0.67 - 2.30) | 0.491 |
| Hemoglobin | 0.84 (0.76 - 0.93) | **<.001** | 0.85 (0.76 - 0.95) | **0.004** |
| IL-6 levels |  |  |  |  |
| G0_IL6_ | 1.00 (Reference) |  | 1.00 (Reference) |  |
| G1_IL6_ | 2.70 (1.46 - 5.00) | **0.002** | 2.14 (0.97 - 4.70) | 0.058 |
| G2_IL6_ | 6.63 (3.76 - 11.70) | **<.001** | 4.86 (2.42 - 9.73) | **<.001** |
| EBV DNA levels |  |  |  |  |
| G0_EBV_ | 1.00 (Reference) |  | 1.00 (Reference) |  |
| G1_EBV_ | 2.16 (1.18 - 3.97) | **0.013** | 2.71 (1.14 - 6.44) | **0.024** |
| G2_EBV_ | 5.81 (3.32 - 10.16) | **<.001** | 9.43 (4.38 - 20.29) | **<.001** |

^§^UICC/AJCC 8^th^ stage system.

⁺Systemic treatment (chemotherapy with or without antiangiogenic drugs) after diagnosing recurrent or metastatic NPC.

Abbreviations: SD, standard deviation; IQR, interquartile range; BMI, body mass index; IL-6, interleukin-6; EBV, Epstein-Barr virus; (r), recurrence.

**Table S3. Correlation between G0-2_IL-6_ levels and clinical parameters (the full cohort).**

| Variables | Total  (n = 319) | G0_IL6_  (n = 153) | G1_IL6_  (n = 85) | G2_IL6_  (n = 81) | P value^*^ |
| --- | --- | --- | --- | --- | --- |
|  |  |  |  |  |  |
| Hemoglobin concentration (continuous, g/dL) | | | | | |
| median (IQR) | 12.90  (11.25, 14.00) | 13.40  (11.90,14.40) | 13.00  (11.70,13.90) | 11.70  (10.20,13.10) | **<.001** |
| mean ± SD | 12.56 ± 2.18 | 13.08 ± 1.81 | 12.61 ± 2.27 | 11.52 ± 2.37 | **<.001** |
| EBV DNA (copies/mL) | | | | | |
| median (IQR) | 996.0  (64.2,13911.0) | 369.0  (28.0,2830.0) | 1730.0  (271.0,14500.0) | 10700.0  (379.0,108000.0) | **<.001** |
| Treatment line(s) of immunochemotherapy 0.892 | | | | | |
| 1 | 231 (72.41) | 112 (73.20) | 62 (72.94) | 57 (70.37) |  |
| ≥2 | 88 (27.59) | 41 (26.80) | 23 (27.06) | 24 (29.63) |  |
| Sex |  |  |  |  | 0.505 |
| male | 245 (76.80) | 114 (74.51) | 69 (81.18) | 62 (76.54) |  |
| female | 74 (23.20) | 39 (25.49) | 16 (18.82) | 19 (23.46) |  |
| Age, years |  |  |  |  | 0.478 |
| < 46 | 159 (49.84) | 71 (46.41) | 46 (54.12) | 42 (51.85) |  |
| ≥46 | 160 (50.16) | 82 (53.59) | 39 (45.88) | 39 (48.15) |  |
| BMI (kg/m2) |  |  |  |  | **0.002** |
| < 23 | 197 (61.76) | 79 (51.63) | 59 (69.41) | 59 (72.84) |  |
| ≥23 | 122 (38.24) | 74 (48.37) | 26 (30.59) | 22 (27.16) |  |
| T stage |  |  |  |  | 0.279 |
| (r) T0-3 | 271 (84.95) | 135 (88.24) | 69 (81.18) | 67 (82.72) |  |
| (r) T4 | 48 (15.05) | 18 (11.76) | 16 (18.82) | 14 (17.28) |  |
| N stage |  |  |  |  | 0.109 |
| (r) N0-2 | 255 (79.94) | 129 (84.31) | 67 (78.82) | 59 (72.84) |  |
| (r) N3 | 64 (20.06) | 24 (15.69) | 18 (21.18) | 22 (27.16) |  |
| Bone metastases | | | | | **<.001** |
| no | 195 (61.13) | 106 (69.28) | 58 (68.24) | 31 (38.27) |  |
| yes | 124 (38.87) | 47 (30.72) | 27 (31.76) | 50 (61.73) |  |
| Liver metastases | | | | | **<.001** |
| no | 240 (75.24) | 129 (84.31) | 65 (76.47) | 46 (56.79) |  |
| yes | 79 (24.76) | 24 (15.69) | 20 (23.53) | 35 (43.21) |  |
| Lung metastases | | | | | 0.485 |
| No | 247 (77.43) | 122 (79.74) | 66 (77.65) | 59 (72.84) |  |
| yes | 72 (22.57) | 31 (20.26) | 19 (22.35) | 22 (27.16) |  |
| Lymph node metastases | | | | | **<.001** |
| No | 210 (65.83) | 118 (77.12) | 46 (54.12) | 46 (56.79) |  |
| yes | 109 (34.17) | 35 (22.88) | 39 (45.88) | 35 (43.21) |  |
| Number of metastasis lesions | | | | | **<.001** |
| 1-5 | 196 (61.44) | 116 (75.82) | 50 (58.82) | 1. 37.04) |  |
| >5 | 123 (38.56) | 37 (24.18) | 35 (41.18) | 51 (62.96) |  |
| Progression disease **<.001** | | | | | |
| No | 193 (60.50) | 120 (78.43) | 45 (52.94) | 28 (34.57) |  |
| yes | 126 (39.50) | 33 (21.57) | 40 (47.06) | 53 (65.43) |  |
| Death |  |  |  |  | **<.001** |
| No | 250 (78.37) | 137 (89.54) | 66 (77.65) | 47 (58.02) |  |
| yes | 69 (21.63) | 16 (10.46) | 19 (22.35) | 34 (41.98) |  |
| Progression-free survival, months **<.001**^&^ | | | | | |
| median  (95% CI) | 28.10  (24.20-33.60) | 33.60  (32.50-NA) | 25.10  (14.5-NA) | 10.70  (8.73, 16.00) |  |
| Overall survival, months **<.001**^&^ | | | | | |
| median  (95% CI) | NR | NR | NR | 19.60  (17.3-NA) |  |

*P values compare the patient characteristics and outcome events using t-test, Mann-Whitney U test, chi-square test, or exact Fisher test depending on whether the variables were continuous or categorical. ^&^Log-rank test.

Abbreviations: G, grade; SD, standard deviation; IQR, interquartile range; BMI: body mass index; IL-6, interleukin-6; EBV, Epstein-Barr virus; (r), recurrence; NR, not reached; NA, not applicable.


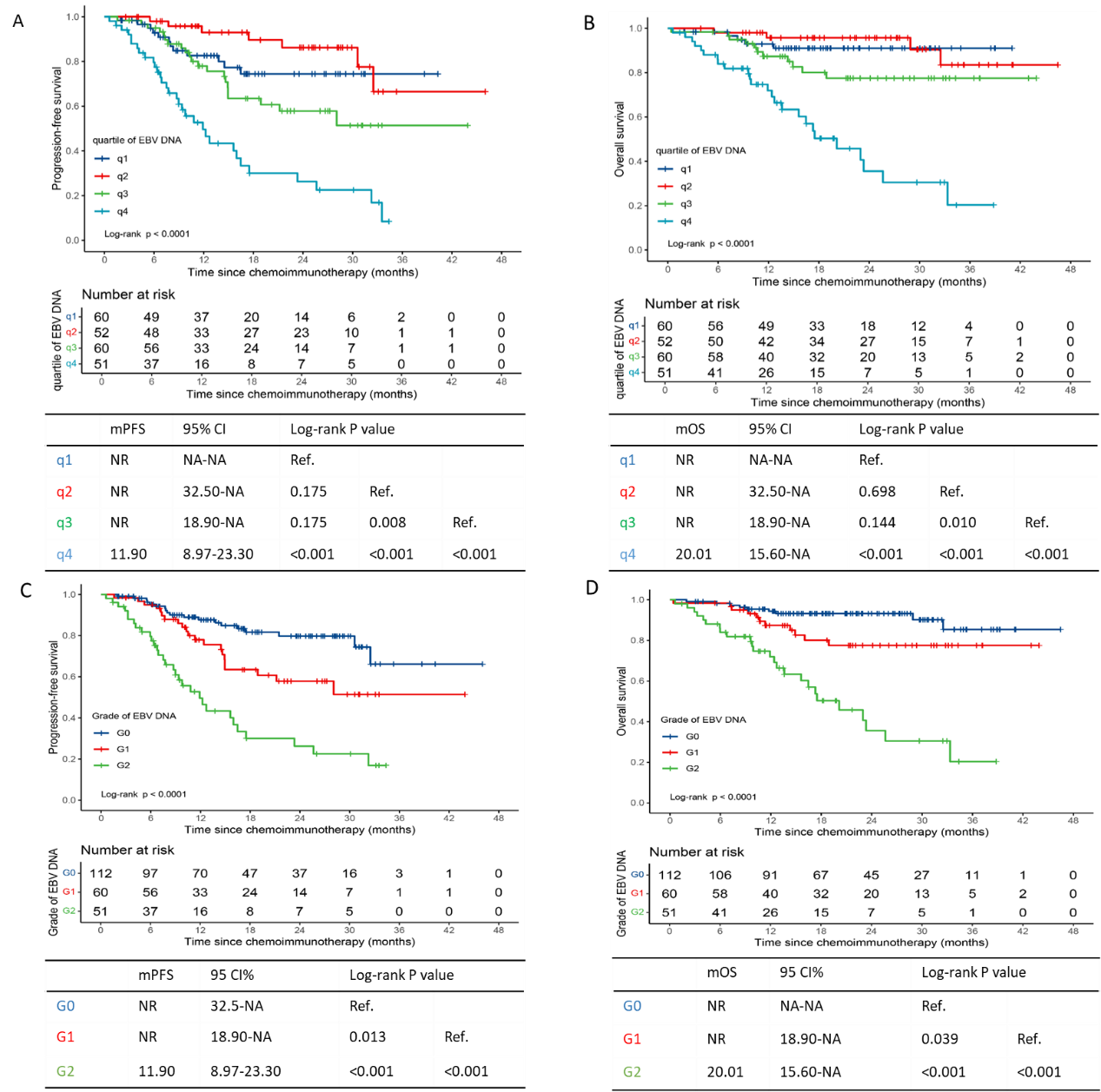


**Figure S1. Survival curves by baseline plasma EBV DNA levels.** Progression-free survival curves for q1-4 _EBV_ levels and G0-2_EBV_ levels (A, C). Overall survival curves for q_EBV_1-4 levels and G0-2_EBV_ levels (B, D) in the training cohort.

Abbreviations: EBV, Epstein-Barr virus; q, quartile; G, grade; NR, not reached; NA, not applicable.

**
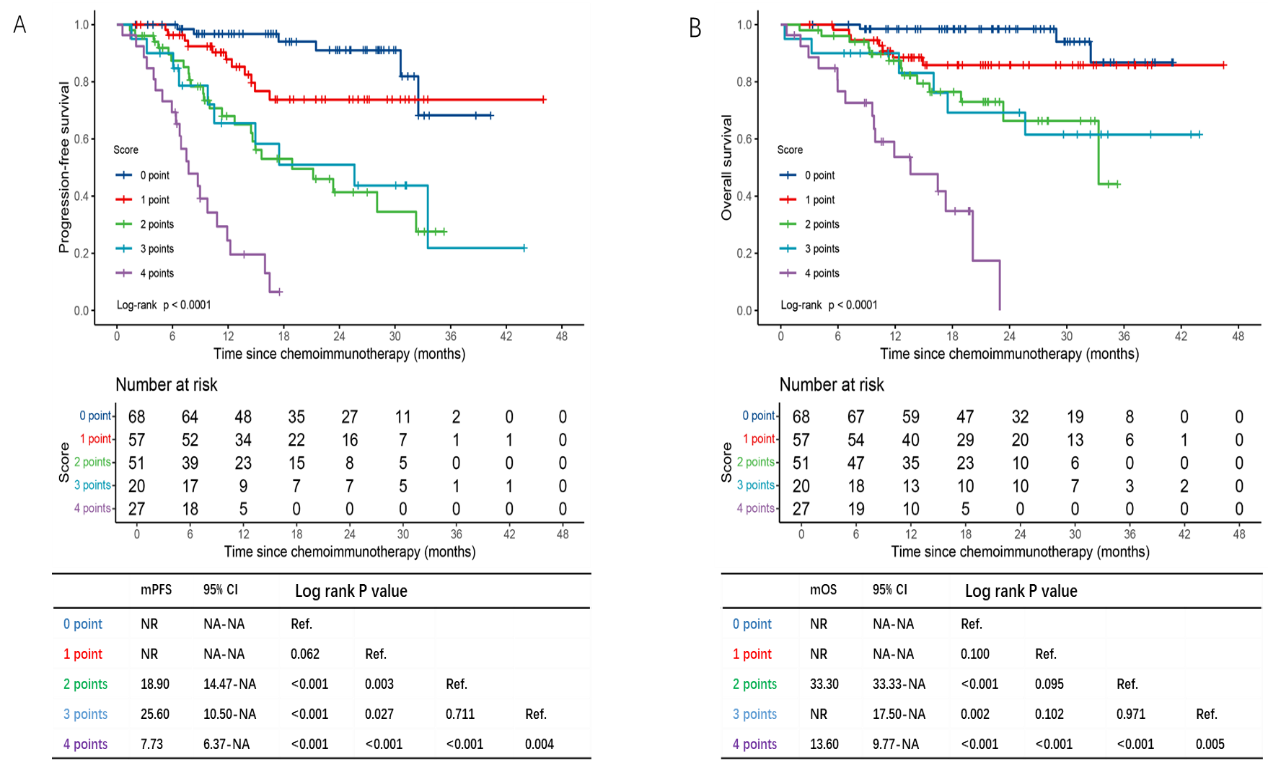
**

**Figure S2. Prognostic scores based on IL-6 and EBV DNA levels.** Kaplan Meier curves of PFS (A) and OS (B) for patients grouped by 0-4 points.

Abbreviations: EBV, Epsteine-Barr virus; HR, hazard ratio; mPFS, median progression-free survival; mOS, median overall survival; NR, not reached; NA, not applicable.

**
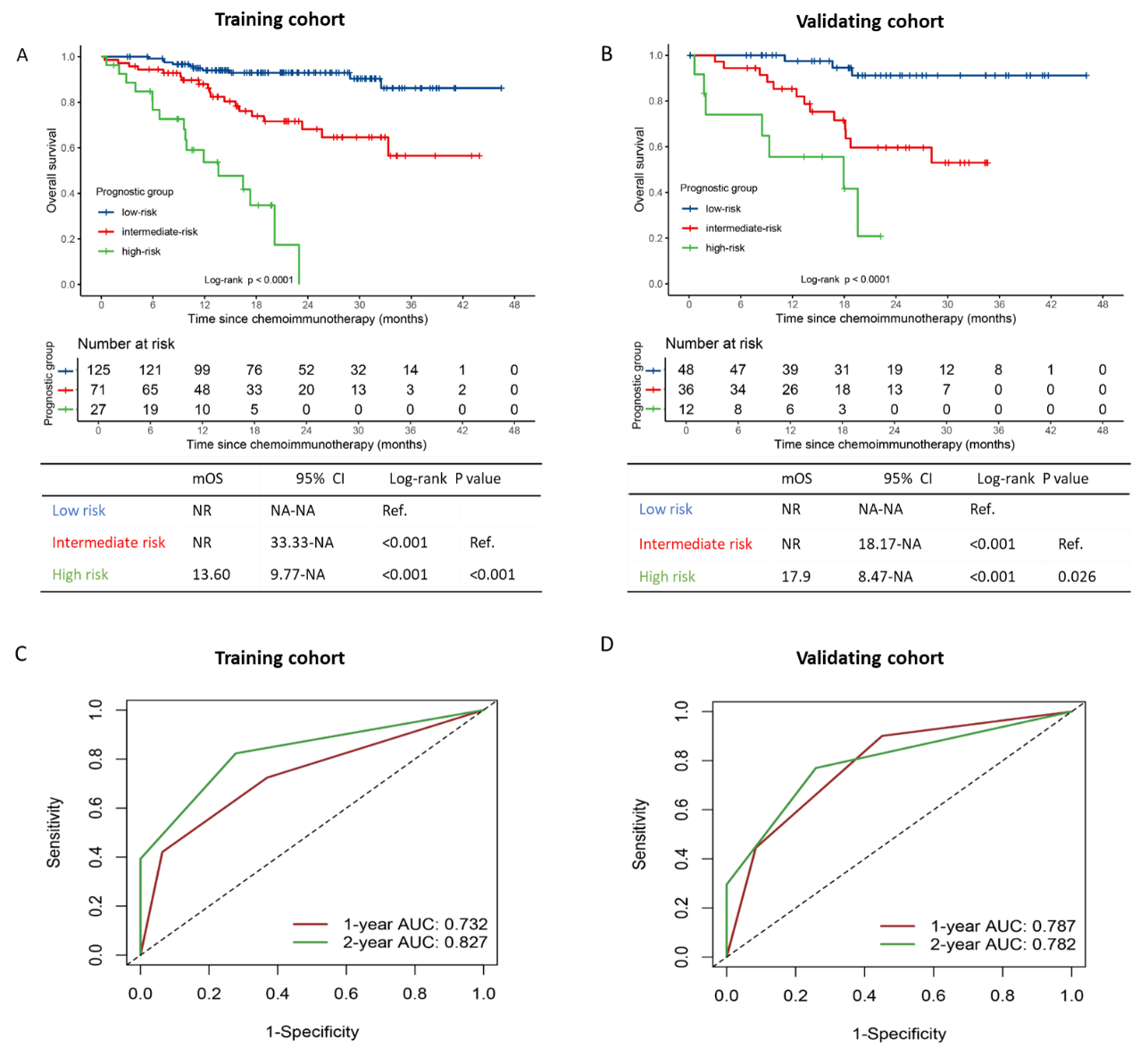
**

**Figure S3. Prognostic risk stratification and model evaluation.** Kaplan Meier curves of OS for patients stratified by different prognostic risk groups in the training and validation cohort (A, B). The time-dependent ROC curves of the prognostic risk grouping for predicting the 1-, and 2-year OS rate in the training and validation cohort (C, D).

Abbreviations: ROC, receiver operating characteristic curves; AUC, area under the curve; OS, overall survival.CI, confidence interval; NR, not reached; NA, not applicable.


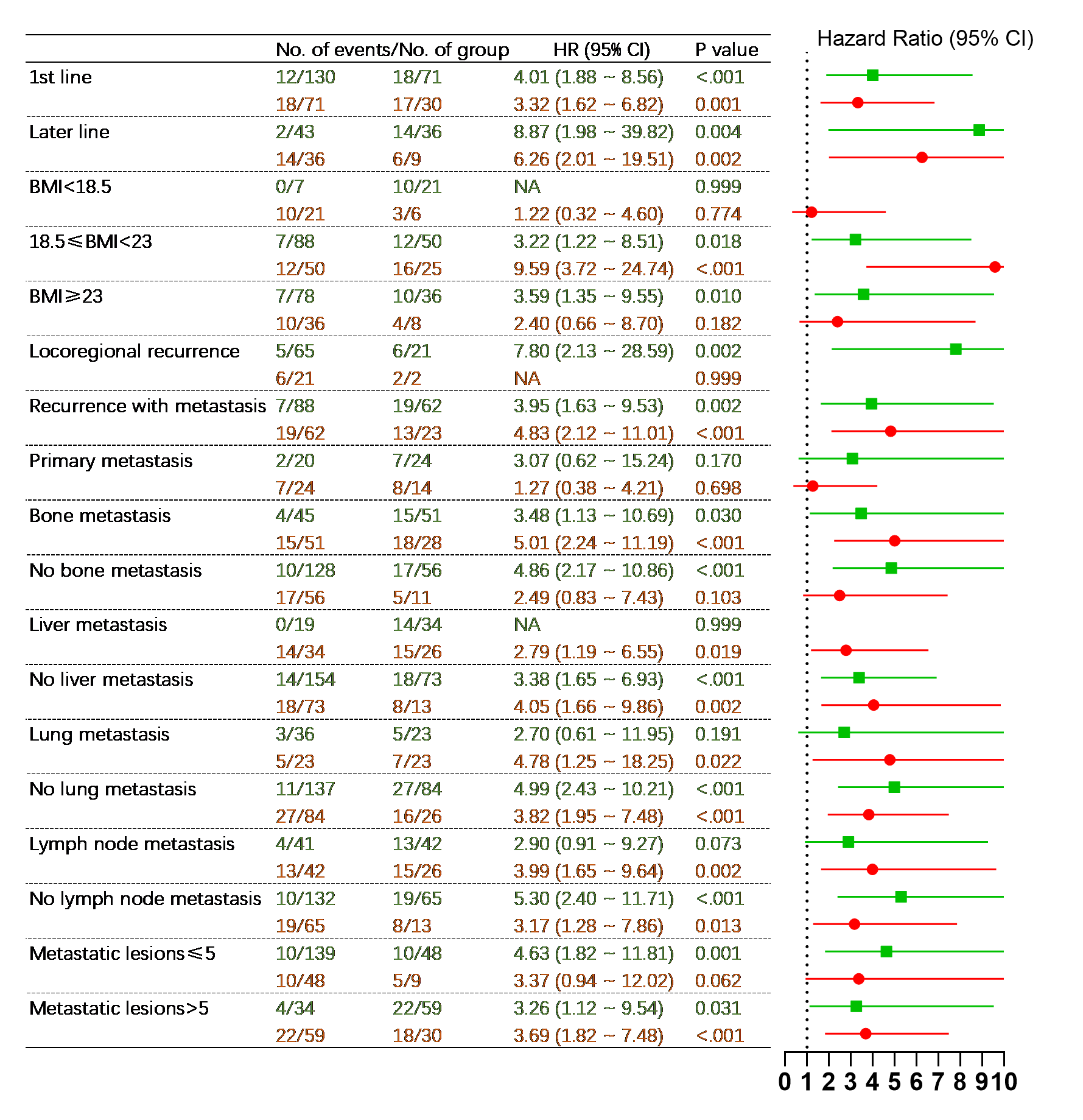


**Figure S4. Hazard ratios for death comparing three prognostic risk groups (low- vs. intermediate-risk in green and intermediate- vs. high-risk in red) in different subgroups of the full cohort (adjusting sex, age, hemoglobin, and chemotherapy regimens).**

Abbreviations: HR, hazard ratio; CI, confidence interval; BMI, body mass index; NA, not applicable.

P values indicated the levels of statistical differences.
